# Becker nevus presenting as pseudofolliculitis barbae

**DOI:** 10.1016/j.jdcr.2026.07.028

**Published:** 2026-07-23

**Authors:** Ameije Anthony, Hannah Jenkins, Matthew Schmeiser, John Moesch, Richard Miller

**Affiliations:** a HCA Healthcare/Morsani College of Medicine GME, HCA FL Largo Hospital, Dermatology, Largo, Florida; b Lake Erie College of Osteopathic Medicine, Bradenton, Florida; c Rocky Vista University, Montana College of Osteopathic Medicine, Billings, Montana; d Georgia Dermatology and Skin Cancer Center, Savannah, Georgia

**Keywords:** adrogen receptor sensitivity, beard distribution, Becker nevus, cutaneous hamartoma, facial Becker nevus, hypertrichosis

## Case report

**Fig 1:**
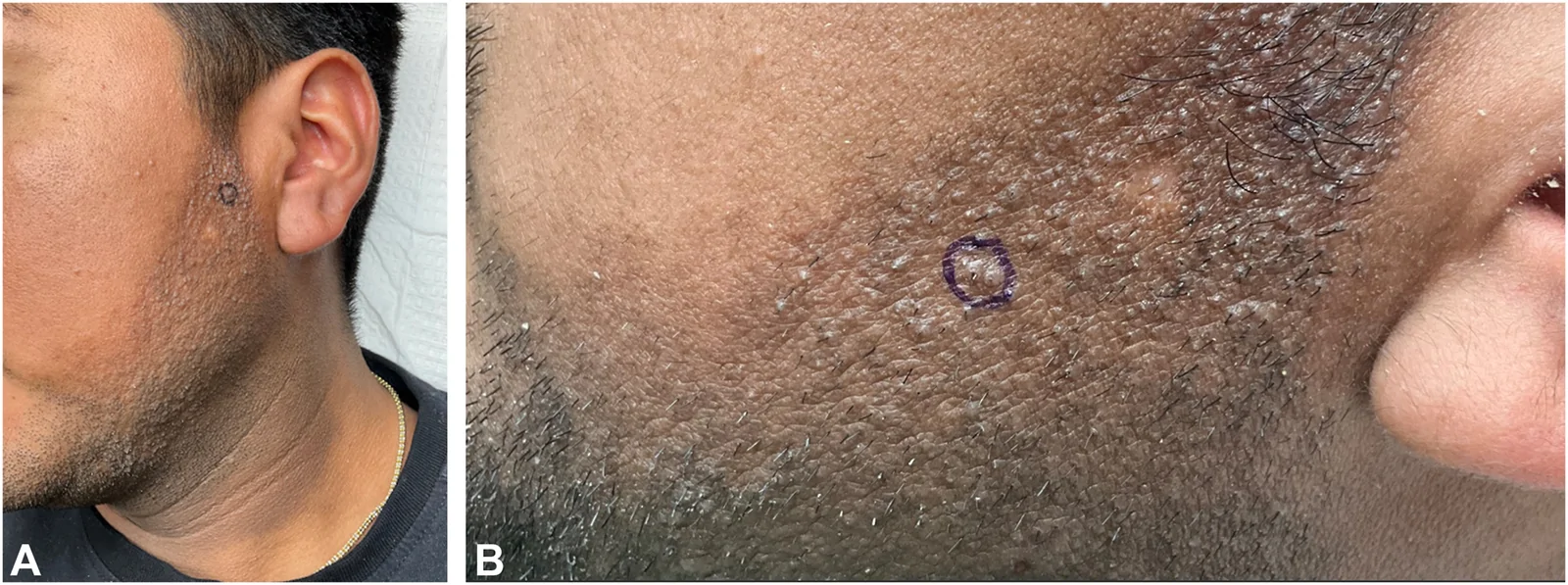
**A,** A hyperpigmented plaque with follicular papules and reported hypertrichosis is visible within the beard distribution on the left side of the facial skin. **B,** The corresponding biopsy site is demarcated with blue ink.

A 25-year-old man presented with complaints of an asymptomatic hyperpigmented plaque with hypertrichosis on the left side of the face in a beard distribution. On physical examination, left-sided hyperpigmentation with increased terminal hair growth was observed, involving the facial and nuchal skin in a beard distribution (Fig 1, *A* and *B*). No muscular or skeletal abnormalities were noted, including absence of breast hypoplasia, scoliosis, or limb asymmetry.

**Question: Which clinical feature most strongly supports the proposed pathogenesis of this condition?**

**A.** Follicular papules.

**B.** Unilateral distribution.

**C.** Hypertrichosis and pubertal onset.

**D.** Basal hyperpigmentation.

**E.** Mild perivascular lymphocytic infiltrate.

## Answer and Discussion

Correct answer: **C.** Hypertrichosis and pubertal onset.

**Fig 2:**
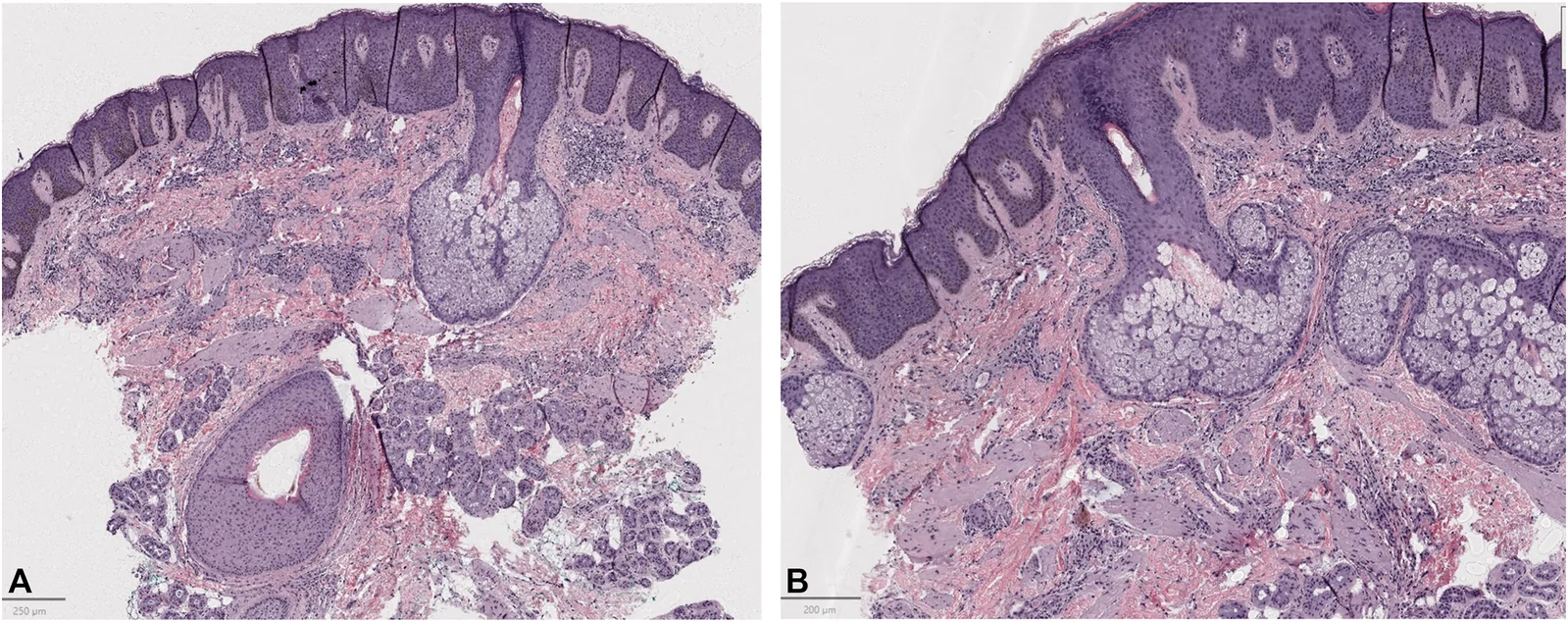
**A,** Histopathologic examination with a low power view demonstrating acanthosis, tabling of the rete ridges, and increased dermal adnexal structures. **B,** Closer view highlighting perifollicular fibrosis, increased basal pigmentation, and dermal smooth muscle proliferation.

The proposed pathogenesis of Becker nevus is due to increased androgen receptor activity or overexpression.^1^^,^^2^ The patient first noticed the lesion at an age of 15 years, and pubertal onset correlates with increased serum androgens. Histopathological examination (Fig 2, *A* and *B*) revealed acanthosis with elongation and broadening of rete ridges with increased basal layer pigmentation. The dermis showed prominent smooth muscle bundle proliferation consistent with a smooth muscle hamartoma, along with associated perifollicular fibrosis and mild perivascular lymphocytic infiltrate. Hair follicles appeared increased in number and size, correlating with hypertrichosis, which is androgen mediated.

Becker nevus is a benign cutaneous hamartoma commonly found on the upper portion of the trunk and upper extremities of men. The diagnosis of Becker nevus is strongly suggested by the simultaneous presence of hyperpigmentation, hypertrichosis, and follicular papules within a plaque. Involvement of the face, particularly in a beard distribution, is rare, with few reported cases in the literature.

Atypical presentation sites of Becker nevus are thought to be related to somatic mosaicism. ACTB mutations have been identified in the affected lesion, while adjacent normal-appearing skin does not harbor the same mutation.^3^ The sharply demarcated borders of this lesion, along with its unilateral presentation, support the idea of somatic mosaicism associated with Becker’s nevus. Our case’s localization to the beard distribution, with associated hypertrichosis during the onset of puberty, further supports the role androgen receptor stimulation plays in lesion development.

Uncommon anatomic locations expand the recognized clinical spectrum of Becker’s nevus and may broaden the clinical differential diagnosis. Medically, there is no indication to treat Becker nevus because management is pursued solely for cosmetic concerns. However, involvement of the beard-bearing skin presents unusual cosmetic and practical considerations. Hypertrichosis in this location may contribute to psychosocial distress and complicate shaving and routine facial hair grooming. Furthermore, conventional laser therapy, which typically combines nonablative fractional resurfacing with laser hair removal, may be less desirable for patients who wish to preserve their beard while reducing lesional hypertrichosis.

This case highlights the importance of considering Becker nevus when evaluating hyperpigmented facial lesions, particularly in the beard area, to guide both appropriate diagnosis and management.

## Conflicts of interest

None disclosed.
